# The Human Right to Water and Unconventional Energy

**DOI:** 10.3390/ijerph15091858

**Published:** 2018-08-28

**Authors:** Robert C Palmer, Damien Short, Walter E. Ted Auch

**Affiliations:** 1 Faculty of Business and Law, Open University, Milton Keynes MK7 6AA, UK; robert.palmer@open.ac.uk; 2 Human Rights Consortium, School of Advanced Study, University of London, London WC1E 7HU, UK; 3 FracTracker Alliance, Cleveland, OH 44120, USA; auch@fractracker.org

**Keywords:** “fracking”, hydraulic fracturing, extreme energy, unconventional energy, right to water, human rights impacts

## Abstract

Access to water, in sufficient quantities and of sufficient quality is vital for human health. The United Nations Committee on Economic, Social and Cultural Rights (in General Comment 15, drafted 2002) argued that access to water was a condition for the enjoyment of the right to an adequate standard of living, inextricably related to the right to the highest attainable standard of health, and thus a human right. On 28 July 2010 the United Nations General Assembly declared safe and clean drinking water and sanitation a human right essential to the full enjoyment of life and all other human rights. This paper charts the international legal development of the right to water and its relevance to discussions surrounding the growth of unconventional energy and its heavy reliance on water. We consider key data from the country with arguably the most mature and extensive industry, the USA, and highlight the implications for water usage and water rights. We conclude that, given the weight of testimony of local people from our research, along with data from scientific literature, non-governmental organization (NGO) and other policy reports, that the right to water for residents living near fracking sites is likely to be severely curtailed. Even so, from the data presented here, we argue that the major issue regarding water use is the shifting of the resource from society to industry and the demonstrable lack of supply-side price signal that would demand that the industry reduce or stabilize its water demand per unit of energy produced. Thus, in the US context alone, there is considerable evidence that the human right to water will be seriously undermined by the growth of the unconventional oil and gas industry, and given its spread around the globe this could soon become a global human rights issue.

## 1. Introduction: ‘Unconventional’ Extreme Energy and Water

This paper examines the development of the right to water in international law and discusses its relevance to a key contemporary social, political and environmental challenge—the growth of unconventional energy. Indeed, in examining the development of the right to water, and its contemporary legal status, we seek to explore the potential impact on the human right to water of a water-intensive unconventional resource extraction industry that could seriously jeopardise people’s ability to realise the right in any meaningful way.

At the outset it is important that we define our terms. How do we define ‘unconventional’ energy? To answer that question, it is perhaps pertinent to explain first ‘conventional’ mineral extraction. In simple terms, it is the extraction of readily available and relatively easy to develop oil and gas from reservoirs trapped in natural geological structures, generally sandstone and carbonate rocks. In the not too distant past, natural geological processes that took place over hundreds of thousands of years provided plentiful hydrocarbon resources. Conventional gas uses traditional methods to extract primary deposits held in underground reservoirs created by the geological processes. Unconventional natural gas resources, such as coal bed methane, tight gas and shale gas are termed ‘unconventional’ because the porosity, permeability, fluid-trapping mechanism, or other characteristics of the strata from which the gas is extracted differ greatly from conventional sandstone, siltstone and carbonate (limestone) sources and are generally much more energy intensive and time consuming to extract. Conventional mineral resources ‘pool’ into convenient reservoirs ready to be exploited, whereas unconventional minerals are present in the entire rock strata. The problem lies therein. In order to extract enough of that unconventional gas, tightly trapped in vast shale plays and coal seams, an amount of extraordinary scientific and engineering developments are going to be required: certainly, it is not a straightforward undertaking. The technique, widely coined as fracking, has been the subject of controversy because of the potential effects that hydraulic fracturing and related oil and gas production activities may have on human health and the environment. The advent of unconventional oil and gas development (UOGD) poses threats to the natural support systems that are necessary for life, all life, specifically air and water. Here we are concerned with perceived threats to the planet’s water resources.

As the easier to extract ‘conventional’ reserves are gradually depleted and global demand for energy rises, there is increasing pressure to exploit so-called ‘unconventional’ energy sources. It is a fact that oil and gas reserves are running out and whilst a small number of oil fields are discovered each year, thus adding to the global reserves, at the same time, ever larger volumes are being extracted that cancel out any new discoveries: we are rapidly reaching what is termed ‘Peak Oil’. Peak Oil is a hypothetical event in time, based on M. King Hubbert’s theory, when global oil production reaches its maximum rate of extraction, after which production will terminally decline. The exact timing of Peak Oil is complex and outside the remit of this paper, but outside estimates places the event between 2020 and 2035, although dates after 2030 have been considered as implausible by some experts [1]. In simplest terms, Peak Oil equates to the world’s oil producers being unable to sustain historical increases in their production: resources will be in freefall. However, demand for oil will remain deep-seated; not just for energy but throughout the entire commodity supply chain of the developed world. UOGD represents a last-ditch attempt to bridge the gap of dwindling oil and gas resources using methods beyond an artificial lift or traditional methods to increase production.

In the simplest sense **‘unconventional gas’, for example,** is natural gas obtained—from secondary deposits—via techniques and methods of production that are, in a given era and location, considered to be new and different. Often these new, unconventional techniques involve ‘stimulation’ processes such as hydraulic fracturing. The International Energy Agency contrasts ‘conventional and ‘unconventional oil’ thus:

“Conventional oil is a category that includes crude oil—and natural gas and its condensates. Crude oil production in 2011 stood at approximately 70 million barrels per day. Unconventional oil consists of a wider variety of liquid sources including oil sands, extra heavy oil, gas to liquids and other liquids. In general, conventional oil is easier and cheaper to produce than unconventional oil. However, the categories “conventional” and “unconventional” do not remain fixed, and over time, as economic and technological conditions evolve, resources hitherto considered unconventional can migrate into the conventional category”.

[2]

A distinction between conventional and unconventional oil and gas extraction needs to be made. Essentially, the equivalent amount of valuable hydrocarbons acquired from conventional wells dwarfs unconventional wells when compared on a well-to-inventory ratio. A simple comparison can be made: a handful of conventional wells are needed to produce the same quantity of hydrocarbons as hundreds of fracking wells situated on scores of well pads.

One could say that as hydrocarbon resources become ever more difficult to exploit, what was once unconventional becomes conventional. There are a number of problems with using such constructed categories as key descriptions in important policy discussions, but they are even more problematic when analyzed conceptually. Michael Klare sought to capture the element of ‘risk’ that was missing with benign terms such as ‘unconventional’ when he first coined the term ‘extreme energy’ to describe the range of relatively new, higher-risk, non-renewable resource extraction processes that have become more attractive to the energy industry as the more easily accessible supplies dwindle and we reach ‘Peak Oil’ [1,3]. However, similar to the term ‘unconventional’, this definition of extreme energy—as a category—is highly problematic as it is dependent upon specific examples; it lacks explanatory or predictive power and leaves open the question of who decides which extractive techniques qualify [4].

A preferable conceptual understanding is extreme energy as a ‘process’, whereby extraction methods grow more intense over time, as easier-to-extract resources are depleted [4]. The foundation of this conception is the simple fact that those energy sources which require the least amount of effort to extract will be used first, and only once those are dwindling will more effort be exerted to gain similar resources. Extreme energy, in this sense, is evident in the history of energy extraction—in the change from gathering ‘sea coal’ from British beaches and exploiting ‘natural oil seeps’, to opencast mining and deep-water oil drilling. Viewed in this light, the concept of extreme energy becomes a lens through which current energy extraction efforts can be explained and the future of the energy industry predicted. Using this extreme energy lens necessitates an understanding of ‘the amount of energy which is needed to obtain energy’, as in this process it is that value which is continually rising. This value may be calculated as either ‘net energy’ or ‘energy return on investment’ (EROI), whereby net energy is the available energy for use after subtracting the energy required for extraction, and EROI is the percentage of energy produced divided by the amount required for extraction. When charted together, the net energy resource available to society is seen to decrease along with EROI in a curved mathematical relationship, which forms the ‘energy cliff’—i.e., the point at which EROI becomes increasingly low and net energy drops to zero [4]. In the extreme energy process, the economic system can be conceptualised as consisting of two distinct segments, the part which is extracting, refining and producing energy (the energy industry) and everything else, which just consumes energy.

What needs to be clearly understood is that the energy industry is in the rare position where the commodity which it produces is also the main resource it consumes. Therefore, as energy extraction becomes more extreme, while the rest of the economy will be squeezed by decreasing energy availability and rising prices, the energy industry’s rising costs could be offset by the rising revenues it receives. The net result will be a reallocation (through the market or otherwise) of resources from the rest of society to the energy industry, to allow the energy industry to target ever more difficult to extract resources [4]. This process is ongoing as easier-to-extract resources are depleted and we loom ever closer towards ‘Peak Oil’ [1,3], with some arguing we have already reached peak ‘conventional’ oil and gas but are yet to reach total peak—which includes potential ‘unconventional’ reserves. Furthermore, data from unconventional extraction methods, such as hydraulic fracturing and tar sands extraction, show that industry is increasingly lurching towards the net energy cliff. Such action on the part of some of the largest and most commercially successful transnational corporations may only be understood as the logical result of the extreme energy process—there simply are no longer enough easier-to-extract ‘conventional’ resources available to meet the needs of growth-driven capitalism [4].

Another issue surrounding the extraction of oil and gas goes beyond the issue of energy usage. Despite coal and gas’s ability to supply a significant amount of our contemporary energy needs, oil is the powerhouse of the global economy. Oil is not simply restricted to providing most of our energy needs for domestic and commercial sectors, among other things it also oils the bearings of industry, binds our roads together and is the principle ingredient in a vast array of materials in society, particularly plastics and chemicals. Accordingly, dwindling supplies will have a detrimental knock-on effect all the way through the global commodity supply chain. Peak Oil and Gas will not only mean an inevitable price hike in energy, commodities, such as plastics, will also become extremely expensive. UOGD—arguably—offers some respite to valuable oil resources that would be better allocated to the production of commodities rather than producing energy. What is problematic is that this possible and, inevitably, temporary solution to offsetting the realities of oil and gas reserves in terminal decline could also bring unacceptable risks to public health and the environment, and even that is leaving aside arguably most important issue of the UOGD discussion—the contribution to greenhouse gasses and climate change of a much less efficient form of energy [4].

**Figure 1:**
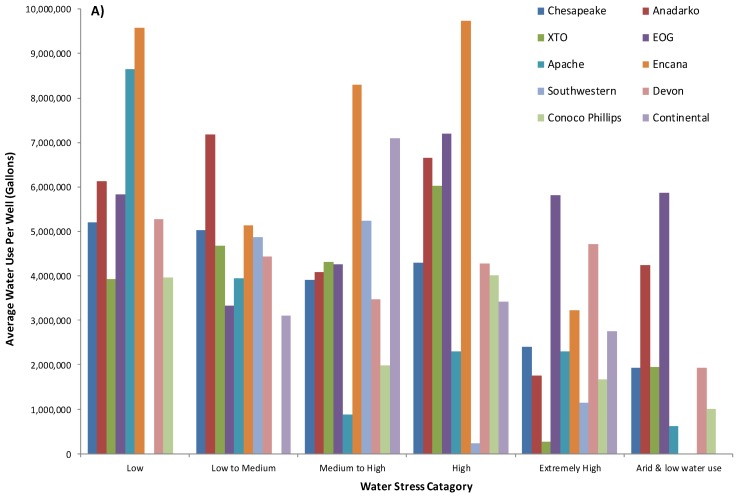

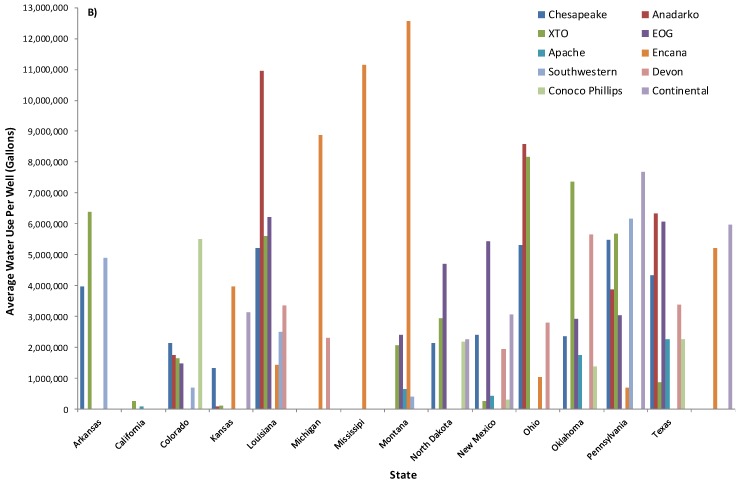
(**A**) Average Hydraulically Fractured Oil & Gas Well Water Demand Per Well across ten companies and six water stress environments and (**B**) fourteen US States and the aforementioned ten companies.

Even so, under the process of extreme energy, one of the most precious resources that is being reallocated away from society to industry is water. Water impacts are one of the most contentious and widely publicized environmental, and as we argue herein, human rights issues, connected with unconventional energy extraction, including but not limited to: groundwater contamination, water use, and contaminated water waste disposal. For example, unconventional gas production is a highly water-intensive process, with a typical single well requiring around 5–11 million gallons of water, and an average well-pad cluster up to 60 million gallons, to drill and fracture, depending on the basin and geological formation [5]. The vast majority of this water is used during the fracturing process, with large volumes of water pumped into the well with 3300–5000 thousand tons of sand (i.e., proppant) and chemicals to facilitate the extraction of the gas; the remainder is used in the drilling stage, with water being the major component of the drilling fluids. Once that water is used by the industry it is no longer a useful resource for society and must be disposed of in what are called Class II Salt Water or Brine Disposal wells. While increasing quantities of water are being recycled and reused in the U.S., freshwater is still used in high quantities for the drilling operations as ‘produced’ water is more likely to damage the equipment and reduce the chance of a ‘successful well’. The industry’s requirements [6] for such quantities of freshwater is clearly a serious concern in water-scarce regions of the world and in places with high cumulative demand for water. Furthermore, the relationship between water demand and ‘produced’ water production is highly correlated across many of the U.S. shale plays with waste production a linear function of water used during the hydraulic fracturing process [7] see Figure 1 (below).

The large quantities of water used by the fracking industry is but one of many serious concerns. The contamination of groundwater sources [8], from failure in the well casing over time [9], what industry refers to as ‘zonal isolation’ failure, is a very serious issue across regions that have seen considerable fracking development to date, and has duly featured as a central public relations battleground for industry and pro-fracking governments. Even so, arguably the most concerning issue with fracking’s use of water is the issue of produced/waste water treatment and disposal often simply referred to as ‘waste water management’. And yet, the risks in this regard go well beyond the concerns of corporate risk minimisation. Indeed, the whole process of dealing with fracking’s waste water is a highly risky business for local populations and the environment with considerable risks of water or soil contamination from surface leaks and spills [10], but perhaps the most concerning issue with waste water is that it can contain significant amounts of radioactive material [11] due to the “naturally occurring hypersaline brines associated with the formations targeted for natural gas production” [12]. For instance, radium has been found to be building up in rivers downstream of shale gas waste discharge points in Pennsylvania [11,12,13]. Vengosh et al. summarised the overall risks posed by fracking development for water as being four-fold [14]:

- Contamination of shallow aquifers in areas adjacent to shale gas development through stray gas leaking from improperly constructed or failing gas wells.
- Contamination of water resources in areas of shale gas development and/or waste management by spills, leaks, or disposal of hydraulic fracturing fluids and inadequately treated wastewaters.
- Accumulation of metals and radioactive elements on stream, river and lake sediments in wastewater disposal or spill sites, posing an additional long-term impact by slowly releasing toxic elements and radiation to the environment in the impacted areas.
- The water footprint through withdrawals of valuable fresh water from dry areas and overexploitation of limited or diminished water resources for shale gas development.

In addition to concerns over groundwater and surface water pollution, other primary concerns include air pollution, greenhouse gas emissions and radiation. Whilst the issue of aesthetics to the environment is often overlooked as a primary concern in the UOGD debate, vast tracts of areas of outstanding natural beauty have been scarred across the United States of America. All these concerns have been exacerbated in the U.S. by the Energy Policy Act 2005 (infamously known as the Halliburton Loophole), which, for example, exempts unconventional oil and gas production and delivery from the Safe Drinking Water Act (SDWA) (Also the Clean Water Act, the Clean Air Act and the Comprehensive Environmental Response, Compensation, and Liability Act (CERCLA)) [15]. In terms of water, the Halliburton Loophole essentially addresses the issue of water quality; however, it is not trite to say that there is a succinct link between water quality and quantity. Palpably, if groundwater and shallow aquifers are polluted/contaminated by hydraulic fracturing and related oil and gas production activities—and no longer viable as a freshwater resource—then water quantity is also diminished. The issue of the right to water encompasses both water quality and quantity: both are essential facets to the right which, in turn, is essential to the ‘minimally good life’ and the realisation of all human rights.

## 2. Status of the Right to Water in International Law

The contemporary legal basis for a right to water at an international level is imprecise and uncertain. This paper, in part, examines the development of the right to water in terms of international law. In examining the development of the right to water, and its contemporary legal status quo, we seek to explore the potential impact of the human right to water through the ‘fracking’ dialogue and the impact of UOGD on people’s ability to realise the right. The connection between UOGD and the right to water requires consideration because the contrast offers a dramatic example of public decision-making diminishing perceived and guaranteed human rights. Here we are concerned with effects of the UOGD industry on water quality and the inequitable situation (for those who live in close proximity of well pads) that arises with regards to not just accessing the vital resource but also having a sustainable and potable supply.

In both human and environmental terms, biology dictates that water—after air—is the most important resource to safeguard human and ecological survival [16,17]. Freshwater is a vital resource for natural ecosystems, human physical and mental health, and various human socioeconomic needs. Many of the contemporary water issues have historical foundations. Indeed, Malcolm Langford refers to ‘millennia-old struggles over the ownership of water, the pollution and depletion of water sources, and conflicting water uses’ [18]. The importance of a global sustainable supply of freshwater is ubiquitous but ‘water resources are under pressure to meet future demands due to population growth and climate change’ [19,20]. Furthermore, it has been argued that the threat posed by worldwide groundwater depletion to global water security is far greater than is currently accepted [21]. According to the United Nations, around 1.2 billion people live in regions of water scarcity, and a further 1.6 billion people live in regions of economic water shortages [22].

The international community has been faced with old threats and new challenges since the onset of the 21st century. In view of this, what is novel is the scale of old problems and impending environmental threats from global warming and, due to advances in technology, far more invasive new industrial practices than have been witnessed in the past, of which UOGD is a prime example. In recent times water scarcity has been the issue that has driven the debate with regards to the human right to water. The hypothesis Curry [23] makes is that a global effort is required to coordinate endeavours to combat freshwater scarcity. In his opinion, the recognition of the right to water (by the UN) ‘would be a building block to initiate the chain of decisions necessary to prevent the dire effects of water scarcity’ [23]. However, in a setting where the introduction of invasive industrial practices threatens both equitable access to water and to devastate water quality, UOGD has added a different dimension to the right to water, which is emerging ever greater as a human right that needs to be recognised at a universal level [24,25].

Whilst there is an impending global crisis with regards to water scarcity, the western world, owing to a perceived abundance of the resource, has maintained an artificial stance and ignores what can be described as an impending crisis [26,27]. This is a precarious situation as even coordinated efforts to combat future global water scarcity are foreseen to fall short of addressing looming human health, environmental and financial crises. Palpably, water scarcity is linked to food insecurity which, in turn, leads to human migration as populations seek to settle in other—more resource rich—geographic locations. Human migration in this context is taken to mean the movement of human populations to settle in other geographic locations, due to any cause other than intended and voluntary movement. In that respect, movement associated with disaster, conflict, or forced migration constitutes human migration. With perceived threats regarding conflict over resources, and the real possibility of ‘Water Wars’ as a consequence of food insecurity and water scarcity, a UN covenant recognising the human right to water ‘will not solve water scarcity by itself, but it will establish the framework necessary for implementing any solution’ [28].

Whilst it is beyond the scope of this paper to give a detailed commentary on human migration and resource conflicts, it is important to highlight associated difficulties that are likely to exacerbate the water scarcity crisis if UOGD continues on proposed sites around the world. Evidently, from the available scientific evidence, the vast quantities of freshwater used in the process will put an unacceptable strain on vital water resources. Unfortunately, the current policy on addressing human migration and resource conflicts is inconsistent with guaranteeing fundamental human rights and freedoms. The Oxford Research Group (ORG) recognised that:

“The current security paradigm adopted by most governments and their defence forces is based on the flawed premise that insecurity can be controlled through military force or containment, thus maintaining the status quo. This has been termed the ‘control paradigm”.

[29]

In human rights terms, the ‘control paradigm’ strikes at the very heart of human dignity, life and health. Within the narrative of the ‘wide range’ of human rights documents, particularly since the 1970s, elements of the right to water are required to be adequate for human dignity, life and health. Indeed, as will be seen below, in accordance with Article 11(1) and 12 of the International Covenant on Economic, Social and Cultural Rights (ICESCR) these three key elements are all-pervading and embody, perhaps personify, the right to water’s normative content in international soft law documents [30]. Curry acknowledged that the right to water, like all other human rights, is ‘derived from a basic acknowledgment of the dignity of all human beings’ [23]. First declared in the Universal Declaration of Human Rights (UDHR), human dignity stands as the ‘minimum definition of what it means to be human in any morally tolerable form of society’ [31]. Curry [23] further argues that a lack or a denial to clean freshwater fails to meet this minimum standard of dignity and that a right to water would ensure that industrial technologies will not take priority over domestic uses. At present it would also ensure that those who disrupt access to clean freshwater are held accountable [23].

It is argued by the ORG that a new approach to security is required that addresses the drivers of conflict: ‘curing the disease’ rather than ‘fighting the symptoms’ [29]. The concept of ‘sustainable security’ is one viable alternative and addresses human rights concerns protected by the UDHR [23]. Perhaps, the Bolivian alternative to the ‘control paradigm’ is the precursor to sensible resource security: Bolivia’s army already have a role in the ‘protection of Mother Earth’ [32]. Whereas there remains a military element to the Bolivian solution, the forerunner to any such action is safeguarding vital natural resources rather than vested industrial interests. Famous for its ‘Law for the Rights of Mother Earth’ [33], where ‘protection of water from contamination’ is a right, Bolivia’s Constitution (2009) also established a precedent by formally recognising water as a ‘basic human right’ [32]. In fact, Article 124 of the Constitution states that citizens who violate the ‘constitutional regime of natural resources’ is recognised as committing an act of treason against the country.

What is noteworthy is that the Bolivian constitution protects human dignity, life and health through giving the environment standing in the courts through its constitution, thus highlighting the inalienable nexus between human and environmental rights. It can be asserted that the expansion of UOGD will impair any attempts at addressing water scarcity and curtail those rights, particularly as the technology will both deplete and contaminate available water resources. Despite Bolivia’s recognition of water as a human right, there have been recent concerns over UOGD and its compatibility with their laws. The issue of whether the technology can be introduced in the country has met with fierce resistance including the matter being discussed in the International Tribunal for the Rights of Nature (December 2014). It can be asserted that, even where progressive developments towards recognising and codifying the right to water exist, UOGD poses a significant threat to human health and the environment as companies such as Halliburton move to exploit Bolivia’s 48 trillion cubic feet of shale gas, which could permanently contaminate 242 billion litres of water and emit 2.6 billion tonnes of carbon dioxide. The question is: will the Law of Mother Nature allow the country to frack rare water resources [34]?

Another major facet to the global water scarcity crisis is an exponential increase in population in the future; thus, owing to the vast quantities of water used in unconventional oil and gas production, the introduction of UOGD will place an insurmountable strain on freshwater resources globally. According to the Population Reference Bureau [35], the world’s population will reach 9.8 billion in 2050, up 31% from the current estimation of 7.5 billion. A major concern is that some of the largest potential oil and gas reserves are in countries that already have a water scarcity issue, for instance, China. However, China’s population (like Europe) is predicted to decline by 2050, albeit there will still be around 1.3 billion people. By 2050, India will be the most populous country in the world (1.7 billion) and has huge potential unconventional oil and gas reserves, particularly in the Cambay basin. With a population of 1.3 billion today, its groundwater resource is already being depleted at an alarming rate. The introduction of UOGD (and 323 million people) will palpably overwhelm the fragile resource. In addition, there is a large UOGD potential in Africa; again, freshwater is at a premium on the continent. However, 30 African nations are expected to at least double in population by 2050. Nigeria, for instance is expected to have the world’s second largest population increase of 220 million people; again, Nigeria has a large UOGD potential with the same threat to freshwater reserves.

Contemporary media coverage and the political backdrop to water scarcity is overshadowed by ‘the impending energy crisis and the search for sustainable solutions’ [23]. UOGD is at the forefront of political aspirations to address the energy crisis and instead of specific steps being taken to produce a sustainable water policy or any relevant technological changes to promote such policies the invasive technology of hydraulic fracturing threatens to be the antithesis to combating water scarcity. In the meantime, weak laws are being watered down by intense corporate lobbying where human rights and environmental concerns are secondary. The balance of lobbying between the wider shareholders and corporate lobbyists has become so disparate that those who pursue human rights and environmental issues from a non-industry perspective are drowned out by the financial might of corporations. Corporations can launch massive offensives to influence issues which put profit above all else; hence, the type of regulation that makes them more competitive. UOGD has been a prime example [36]. The ‘perpetuation of ignorance’ surrounding water scarcity will seemingly create human rights abuses owing to the disastrous circumstances associated with it; such as, water-related diseases, famine, drought and inevitable fatalities to humans, flora and fauna [36].

This paper advocates the growing consensus that if UOGD became widespread in the western world and beyond, then the escalating water scarcity issues associated with the technology could be catastrophic. Human rights protagonists have sought to ground the right to water in international law in the decades following the ICESCR but renewed attempts to establish the human right to water have been plagued by a ‘lack of legal articulation’ ever since [37]. Whilst some interpret the right as lacking ‘an explicit and comprehensive expression in international human rights law’ and therefore does not exist in that context [37], it can still be derived—albeit within a limited scope—from the explicitly protected rights recognised within the ICESCR. Nevertheless, such a derivation is not without criticism, as will be seen in the next section [38,39].

According to its charter, the United Nations has succinct purposes including; maintaining international peace and security; developing amiable relations among nations; and cooperating in solving international economic, social, cultural and humanitarian problems. In addition, it seeks to promote respect for human rights and fundamental freedoms and to be central to coordinating the actions of members in reaching such ends. These aims were reaffirmed and clarified in the 2000 United Nations Millennium Development Goals (MDGs) which were expected to be achieved by the year 2015; one of those goals was to eradicate extreme poverty and hunger. Although the issue of whether the MDGs were achieved by 2015 is outside of the scope of this paper, realising a human right to water is fundamental to the eradication of poverty and hunger. Accordingly, it is questionable whether the introduction of UOGD and the control paradigm safeguard the raison d’être of the UN and strive to attain MDGs in the future.

On the surface, the right to water ‘has been recognised in a wide range of international documents, including treaties, declarations and other standards’ [40] but the reality is such that water remains unrecognised explicitly as an autonomous human right in international treaties, although international human rights law involves specific obligations relating to access to safe drinking water and sanitation [41,42,43,44]. Whilst one could say that the right ought to be derived from numerous human rights documents as they are intended, an explicit right to water has failed to be adopted by states universally [45]. This is perhaps why the overall theme within recent literature suggests that the right to water has been a ‘latent component’ of other socioeconomic rights contained within the ICESCR, other international human rights treaties and other water-related treaties [37]. For instance, in 2001 the European Committee of Ministers recognised that the rights to be free from hunger and the right to an adequate standard of living that are contained within international human rights instruments include the right to a minimum quantity of water of satisfactory quality.

The summary of the development of the right to water that follows will demonstrate that the intentions of the Committee on Economic, Social and Cultural Rights (CESCR) has been to ‘articulate a pre-existing right’ [37]. Moreover, despite the existence of a prior or contemporary autonomous right being disputed, it can be demonstrated that the right has a firm legal standing, particularly when supported by environmental law, international water law and state-related jurisprudence. This is evident in a wide range of national legal instruments which contain state duties/obligations and entitlements of citizens with regards to, among others, access to water and sanitation.

Fundamentally, the right to water—at an international level—resides in Articles 11 and 12 of the ICESCR 1966. Thus far, since no intergovernmental organisation enjoys exclusive responsibility for water resources, the choice of the appropriate law-making forum falls to governments [38]. Constitutional provisions can be put into operation in three principal ways: development of legislation, enforcement in courts, and in political discourse. There are absolutely no guarantees—legal or otherwise—that the insertion of access to water in a constitution will lead to its inevitable implementation. However, establishing the right to water and sanitation within constitutions and a series of state obligations that create the necessary legal, social and economic conditions represent a step towards ensuring the realisation of the right. Importantly, many national constitutions impose specific duties upon the state to ensure availability, quality, and accessibility for their citizens [36]. Kenya, for example, has included in its constitution that every person has ‘the right to water in adequate quantities and of reasonable quality’ and that ‘every person has the right to a reasonable standard of sanitation’; thus, both the right to water and sanitation have been enshrined in law [46].

## 3. The Development of the Right to Water

At the national level, despite the absence of a ubiquitous right, the right to water and sanitation has been progressively more recognised in constitutions, legislation and courts globally [46,47]. Some countries have broad provisions addressing not just the quantity of drinking water, but the quality of water and sanitation services holistically; however, universality is far from being achieved. It is noteworthy that most water laws that been adopted since General Comment No. 15 (below) and that are currently being drafted (or under revision) contain provisions based on the human rights dimension of access to water. For instance, the Uruguayan 2004 referendum based on a constitutional amendment regarding public ownership of water supply and water and sanitation provisions was supported by two-thirds of the population. Subsequently, the constitution of Uruguay has been amended and now stipulates that ‘[a]ccess to drinking water and access to sanitation constitute fundamental human rights’.

The origins of the ‘right to water’ can be traced to 1946 when, whilst adopting its constitution, the World Health Organization (WHO) declared that ‘the enjoyment of the highest attainable standard of health is one of the fundamental rights of every human being’ [23]. In 1948, with the adoption of the UDHR, Article 25 used similar language as the WHO two years earlier and bound the right to an adequate standard of living with the right to health. The right to water was not addressed in either document. Reminiscent of the ICESCR two decades later, which also omitted water, we can deduce that many of the interpretive issues that have curtailed the development of the right to water do not merely originate from these early omissions but have continued in the same vein [23]. However, a continual stream of declarations and treaties has followed the ICESCR; it is notable that many of them have attempted to realise the right over the decades.

In 1977, the United Nations Water Conference in Mar del Plata, Argentina [48], first established the concept of basic water requirements to meet fundamental human needs. Its Action Plan asserted that everyone has the right to have access to drinking water in a quantity and quality that meet their basic needs. There are three human rights treaties that unequivocally declare ‘water’ as a right: The Convention on the Elimination of All Forms of Discrimination against Women (CEDAW) [49], the Convention on the Rights of the Child (CRC) [42], and the Convention on the Rights of Persons with Disabilities (CRPD) [43]. In 1979, the Convention on the Elimination of All Forms of Discrimination against Women (CEDAW) was signed (adopted 1981). Article 14(2) (h) (CEDAW) specified that states shall ensure women the right to ‘enjoy adequate living conditions, particularly in relation to … water supply’ [49]. A decade later the CRC was adopted; again, the CRC explicitly referred to water within its text [42]. Whilst the provisions of the ICESCR fail to mention water, both the CEDAW and the CRC mention the human right to water, as part of the right to development (CEDAW) and in relation to a universal right to health (CRC). In the context of opposing UOGD, the CRC is of vital importance because it imparts children with the right to clean drinking water free from the ‘dangers and risks of environmental pollution’ [42].

The Mar del Plata Conference [48] was established when ‘Agenda 21’ was adopted at the United Nations Conference on Environment and Development in June 1992 (Rio) [50]. ‘Agenda 21’ denoted as the ‘Programme of Action for Sustainable Development’, included a separate chapter on freshwater resources (Chapter 18). Chapter 18 endorsed the Resolution of the Mar del Plata Water Conference that all peoples have the right to drinking water whilst naming this principle ‘the commonly agreed premise’ [48]. Again in 1992, the Protocol on Water and Health to the United Nations Economic Commission for Europe’s ‘Convention on the Protection and Use of Transboundary Watercourses and International Lakes’ sought to protect water resources (used as sources for drinking water) by ensuring states take appropriate measures to prevent them from being polluted [46,47].

Another important development in 1992 came in the guise of the Dublin Statement [51]. Of note, the statement advocated—in Principle One—a holistic approach linking ‘socio-economic development with environmental protection’ when addressing effective resource management [38]. 

Whilst it is outside the remit of this paper, it is important to note that the Dublin Statement introduced the issue of ‘affordable price’ into the development of the right to water. Principle 4 of the statement stated that ‘it is vital to recognise first the basic right of all human beings to have access to clean water and sanitation at an affordable price’. In terms of that development, pricing water has both its merits and disadvantages e.g., culturally it would be contrary to some cultures to pay for water whereas other capitalist cultures would demand a price for the resource (but some would argue that capitalism itself is the antithesis to an autonomous right to water) [52]. That said, the Dublin Statement brought to the fore the inalienable nexus between human rights and environmental rights, which is essential to recognise within the UOGD debate, particularly because provisions on the right to enjoy a healthy environment (formally adopted in most constitutions in 1992) may afford a legal basis for the improvement of water quality, for instance, through the prevention of pollution and the provision of adequate sanitation. Integrated water resource management (IWRM) has been developed to allocate water with reference to efficiency and sustainability and is defined as ‘the co-ordinated management of water, land and related resources with a view to maximising socio-economic welfare in an equitable manner without compromising ecosystem sustainability’ [38,53,54].

Whilst it is unclear how a human rights approach to water as a vital ‘socio-economic’ resource and an environmental rights approach to water as an ‘environmental’ resource can be reconciled, the UOGD debate necessitates the adoption of human rights and environmental law because the technology poses significant risks to both human health and the environment. Accordingly, fundamental environmental principles and fundamental human rights and freedoms can be utilised in tandem to secure the right to water. Tully correctly recognised an example of the possible affiliation/homogenisation stating: ‘that environmental legislation which employs pollution abatement schemes and economic incentives for water conservation or recycling could usefully complement a human rights framework’ [39]. Dublin was, in effect, a preparatory meeting for the Rio Summit later in 1992 [52]. At that summit Agenda 21 referred to as the ‘Programme of Action for Sustainable Development’. In particular, Chapter 18 of the Agenda (water resources) not only endorsed the principle of UN Water Conference in Mar del Plata (that all peoples have the right to water) but affirmed the principle as a ‘commonly agreed premise’ [52,55].

In 1994, within the Programme of Action of the International Conference on Population and Development, states affirmed the right to an adequate standard of living for citizens and their families, including adequate food, clothing, housing, water and sanitation. In 1995, the General Assembly adopted the United Nations Principles for Older Persons. In paragraph 5 the Committee referred to the ‘basic rights’ of ‘access to adequate food, water, shelter, clothing and health care’. In Paragraph 32 the Committee affords those basic rights whilst attaching ‘great importance’ to the principle of ‘Independence’, which ‘demands for older persons the rights contained in Article 11 of (ICESCR 1966)’. In 1996, the United Nations Conference on Human Settlements (Habitat II) adopted the Habitat Agenda; again, water and sanitation were recognised as part of the right to an adequate standard of living. Paragraph 11 of the Agenda is noteworthy because it comprises Article 11(1) verbatim, except for adding the words ‘water and sanitation’.

In 1997, the ‘Water Convention’ coined the concept of ‘vital human needs’ [56], which the International Law Association defined as ‘waters used for immediate human survival, including drinking, cooking, and sanitary needs, as well as water needed for the immediate sustenance of a household’ [57]. Peter Beaumont denotes drinking water as the ‘most’ vital of human needs [58], which is important in this context, particularly as the phrase is a shorthand expression for the ‘minimum core of the human right to water’ [37]. The convention entered into force in 2014 and according to the ‘statements of understanding’ concerning the convention and contained within the *Report of the Sixth Committee convening as a Working Group of the Whole* are comments stating that ‘In determining “vital human needs”, special attention is to be paid to providing sufficient water to sustain human life, including both drinking water and water required for production of food in order to prevent starvation’ [59]. According to McCaffrey, the convention’s provision on vital human needs ‘is consistent with the human right to water’ [17]. The convention, unlike the erstwhile soft law provisions that provide for the right to water, is binding on ratifying states and consequently can be used directly as a normative source of the human right to water and thus provide ‘supportive legal authority for General Comment No 15’ (see below) [36].

In 2000, the UN General Assembly resolution on the Right to Development [60], saw the ‘strongest and most unambiguous’ [55,61] statement recognising a human right to water to date. The resolution affirmed that ‘the rights to food and clean water are fundamental human rights and their promotion constitutes a moral imperative both for national governments and for the international community’. Also, in 2000, General Comment No 14 (CESCR) on the right to the highest attainable standard of health stressed that the drafting history of the ICESCR (particularly the wording of Article 12(2) ICESCR) accepted that the right to health extended to the underlying elements of health, including access to safe drinking water and sanitation [55,62].

In 2002, General Comment No. 15 (GC15) of the UN Committee on Economic, Social and Cultural Rights (CESCR) gave the most complete and persuasive interpretation of the human right to water thus far and represents the ‘first instance’ of a UN body explicitly signifying that the right to water is contained within the ICESCR [23,63]. GC15 declared that the ‘human right to water is indispensable for leading a life in human dignity’ and ‘entitles everyone to sufficient, safe, acceptable, physically accessible and affordable water for personal and domestic uses’. The scientific evidence, that is the basis for objections to UOGD, raises clear-cut questions whether the intentions of the committee are to be realised.

The committee set out that States must ‘adopt effective measures to realize, without discrimination, the right to water’. The notion of discrimination in the context of GC15 is important in the UOGD debate as the technique is not possible in all locations, owing to geological conditions. Only those who live in areas where unconventional oil and gas extraction methods can be adopted are under threat; hence there is a succinct element of discrimination surrounding the issue based on location. Within the normative content of the right to water and the entitlements and freedoms it contains, one such entitlement includes the right ‘to a system of water supply and management that provides equality of opportunity for people to enjoy the right to water’ [23,63]. Again, the geological nature of UOGD prevents equality of opportunity under the umbrella of the right. The right’s normative content ensures that water (and water facilities/services) must be accessible to all without discrimination; therefore, if a legal industrial practice prevents equitable access to water it is in contravention of the right to water. Alternatively, a freedom of the normative content of the right is ‘the right to maintain access to existing water supplies necessary for the right to water, and the right to be free from interference, such as the right to be free from … contamination of water supplies’ [64]. Accordingly, the normative content of the right to water should protect people and the environment from a highly risky and contaminating technique that uses huge amounts of water and highly toxic chemicals.

It is noteworthy that General Comment 15 (GC15), which addresses interpretive issues arising from ICESCR, has become the source of debate that denies ascending the human right of water into a universally binding right. Nonetheless, the language across the various documents of international human rights law is, in the main, consistent. Prior to the adoption of GC15, international human rights documents that either implied or explicitly referred to the human right to water were inclined to vary slightly in their description, but the language was along the same lines [63]. Whilst the comment should not be understated, it has proved problematic because it has been criticised for not having a succinct definition [38]. Langford observed, ‘it is one thing to recognise a human right, it is another to define what it means’; but, on the other hand, the comment elaborated the normative content of the right under the ICESCR [18].

It has been reasoned that weaknesses in GC15 (based upon Articles 11 and 12 of the ICESCR) ‘indicates deficiencies within the content of the standard’ of the right to water [65]. Without a succinct definition, concerning various facets of the right to water, the perceived ‘interpretive creativity’ [37,38] of the CESCR has raised questions concerning whether the right exists. In a damning article by Dennis and Stewart, the committee is accused of being ‘aggressive’ in its effort to ‘deconstruct’ the right to an adequate standard of living, as laid down in Article 11 (food, water, clothing, and housing) [39]. By this means, they assert that the committee ‘has overridden the decisions of the negotiators and taken positions inconsistent with the views of states’ [39]. However, Gleick observed (in notes from the original debate through the construction of the UDHR) that the provisions for food, clothing and housing were not intended to be all-inclusive, but representative or indicative of the ‘component elements of an adequate standard of living’; such standards can, and indeed have, change over time [66].

What is, perhaps, most damning to the recognition to the right to water is Dennis and Stewart’s accusations that the committee rewrote Article 11 ‘by resurrecting and adopting alternatives that were considered and rejected by the negotiators’ [33]. In Tully’s opinion, the committee took an approach which ‘undermines the principle of legal security by reading into a legal text a content which simply is not there’. Dennis and Stewart go further and proclaim that the source of a *separate* right to water is ‘virtually without precedent’. In order to justify that assertion, they refer to the CRC as the ‘only international human rights instrument that even mentions water’ and that they are unaware of any ‘mention of water in the negotiating histories of the UDHR or the ICESCR’ [33]. Evidently, such an insight is erroneous in light of the documents highlighted above, particularly when the Legal Resources for the Right to Water and Sanitation report is considered [31].

Dennis and Stewart’s and—subsequently—Tully’s denial of the longstanding existence of the right was challenged again in the Convention on the Rights of Persons with Disabilities (CRPD)—Article 28 (2) (a) enacted to ensure equal access by persons with disabilities to clean water services, and to ensure access to appropriate and affordable services, devices and other assistance for disability-related needs’ [39]. One of the human rights treaties that explicitly refers to water as part of a protected right, the CRPD came later than their articles and refers to ensuring equitable access to clean, affordable water. Accordingly, despite the objections of such academic commentary, key actors within the various UN committees outwardly disagree when both drafting new conventions and interpreting past treaties; the right to water, for all intents and purposes, is enshrined in human rights discourse and continues to be incorporated in new treaties.

It is evident that the development of the right to water is chronicled within the documents mentioned herein and culminated in the adoption of the 2010 UN General Assembly Resolution on the Human Right to Water and Sanitation [67]. The problem is that that resolution has muddied the waters somewhat, as the plenary sessions suggest. We can only speculate regarding why certain vested economic interests continue to oppose a universal recognition of the right. Despite any judicious read-through of the treaties and documents suggesting that the right does exist and should be recognised, those vested interests impinge on the development of the right to water. In the face of such objections, the question as to whether the right will become autonomous is set to continue. Cullet reasons that, generally speaking, the different developments that have emerged over the past couple of decades have elevated the right to water ‘to a principle of international custom’ [16,68]. What may be confusing, therefore, to the layman, activist or, perhaps, policymaker in a search for redress against UOGD, is that the human right to water is seemingly recognised in international human rights treaties but, at the same time, it is rarely recognised as a legal tenet or enforced: when individuals or groups seek protection they find that access to justice is often fraught with insurmountable difficulties.

The reality is that attempts to establish the right to water, based on the ICESCR alone, have proven slow and controversial [37]. Nonetheless this should come as no great surprise considering the original negotiations are, overall, beyond incarnate recognition. Negotiations that took place in the 1950s and 1960s are incongruent with contemporary scientific knowledge and modern societal needs: the right to water has developed in line with human development (including population increase), scientific advancements and the advent of globalisation. It can be seen as peculiar, owing to the immediate link between water and life, that legal instruments have only been giving increasing importance to the right to water over the past two to three decades. Alternatively, this development is a response to new knowledge and better awareness concerning a set of environmental threats with relatively recent foundations.

The impending—potentially catastrophic—threats with regards to water scarcity, particularly from heavy industry, require international action. UOGD is a matter in question here; certainly, the industry is an environmental threat on numerous levels and (despite industry misdirection and media misinformation regarding its actual age) the way the various methods and scale combine effectively make it ‘a new technology’. In consideration that this type of oil and gas production only became operational in the 21st century, it is accurate to assert that it is still being developed and the ‘manual’ is still being written. UOGD has not, and never will be, a steady-state operating procedure; it will never be perfected. This is largely why the regulatory framework cannot respond to the new science: comprehensive, bespoke guidelines are required to regulate the technology.

To deny the development of the right to water owing to the objections of historic negotiators is, at best, shortsighted, at worst, ignoring the raison d’être of the United Nations and the fact that laws themselves develop. An important but modest question is lost within the relevant literature that needs to be addressed: why was the right to water omitted from the ICESCR? The omission can be one of three things: a drafting oversight; a deliberate omission; or it was considered and rejected by the original negotiators. Most of the contemporary literature is concerned with trying to find ‘novel’ ways in which to find the right from within the wide range of documents (above) or alternatively to deny the right existed in the first place [38]. There is a viewpoint that it was a deliberate omission, which suggests the original drafters (ICESCR) took it for granted that the right would be recognised in the provisions. Indeed, Tully revealed that water was *deliberately* omitted by the drafters ‘as an explicit right on account of its nature: like air, it was considered so fundamental that its formal inclusion was unnecessary’ [38,66].

It has been argued that the ‘intrinsic link with life’ means that water is included as a right ‘in any human right instrument or bill of rights’, regardless of whether or not it is formally incorporated within the list of recognised rights [16,66]. There is a degree of rationalisation when water’s vital role is considered; unquestionably, academic commentary and the CESCR have been frustrated by attempts to deny the right. Bulto observed that ‘the absence of a comprehensive guarantee for the human right to water in the universal human rights treaties has variously been dubbed “odd,” and “startling”’ [36] and, in light of prior General Comments and international instruments adopted in earlier decades containing references to the right (the CESCR endeavoured to break new ground by unequivocally affirming that the International Covenant on Economic Social and Cultural Rights (ICESCR) contains provisions that implicitly contain an autonomous human right to water [36]) the CESCR were responding to being ‘confronted continually with the widespread denial’ of it by states. The committee’s role must be borne in mind; they must consider reports submitted by UN member states on their compliance with the ICESCR, and thus perceived difficulties must have been central when the committee convened for GC15.

Undeniably, one is immediately struck by the fact that both Articles 11 and 12 (and the covenant) are devoid of a separate right to water or the term ‘water’; this omission has in turn created legal and political uncertainty, thus limiting the development and legal status of the right to water. When read in context, however, such uncertainty can be viewed as being overly doctrinaire because good sense and sound judgment would advocate that including the term ‘water’ would be superfluous to the requirements in the covenant at that time.

Certainly, the content of ICESCR Article 11 demands a right to water in order for the right to ‘adequate living standards’ to be achievable. Furthermore, in recognising a ‘fundamental right of everyone to be free from hunger’, water would be essential for that right because crops need water, water is needed for cooking, and livestock need to drink. States would be useless in their obligations to disseminate knowledge ‘of the principles of nutrition’ or ‘developing or reforming agrarian systems in such a way as to achieve the most efficient development and utilisation of natural resources’ without safeguarding water resources.

The wording of Article 11(1) is pivotal here. It states:

“The States Parties to the present Covenant recognize the right of everyone to an adequate standard of living for himself and his family, including adequate food, clothing and housing, and to the continuous improvement of living conditions”.

[30]

The development of the right to water is lost in an interpretive minefield that first began, over seventy years ago. It is clear from the onset of any UN movement towards recognising the right to water that an etymological absurdity has developed alongside it, which, in turn, has weakened its legitimacy as a self-standing human right. This linguistic paradox can be seen to revolve around the term ‘food’. The Concise Oxford Dictionary defines food as ‘any nutritious substance that people or animals *eat or drink* or that plants absorb in order to maintain life and growth’. Certainly, water is defined as a nutrient and is an indispensable ingredient in many mainstay foods—essential to human survival—across the globe. The biological and chemical descriptions that define water as a foodstuff are beyond our remit but as a ‘nutritious substance that people eat or drink … in order to maintain life and growth’ water can be defined as food for the purposes of Article 11. The right to food is recognised in the 1948 Universal Declaration of Human Rights (UDHR) as part of the right to an adequate standard of living, and is protected, as a right, by the ICESCR 1966. Palpably, there is an anomaly that requires reconsideration if all human beings have the right to adequate food and the right to be free from hunger but have no right to adequate water or the right to be free from dehydration [69].

The interpretation of the ICESCR has become central to the development of the right to water within the literature. Bulto, in particular, pays specific attention to the manner in which the CESCR ‘read in’ the right from the implicit terms of Articles 11 and 12 of the ICESCR [37]. This interpretive approach—the purposive approach—that the committee undertook during GC15 has been unceremoniously criticised by the likes of Tully and Dennis and Stewart, despite both methods being universally accepted methods of interpretation [38,39]. In an attempt to prevent interpreting the ICESCR in a narrow and restricted sense, the committee sought to elucidate the purpose of the legislation to reduce ambiguities in a manner that best serves the object and purpose of the covenant. The most visible criticism for using this approach is the way the right to water has been derived from succinct guaranteed human rights. However, those criticisms seemingly originate from the committee’s (again perfectly acceptable legal method) interpretation of the word ‘including’ in Article 11(1). Preceding the list of rights mentioned in the ICESCR, the committee’s reading of the article was such that those rights were ‘not intended to be exhaustive’ [63]. Using this rational (again accepted) interpretative method, Curry recognised that ‘since the right to water is a natural extension of those rights listed in the ICESCR, recognition of the right to water is as essential as all others mentioned’ [23].

An additional factor with regards to the content of the right to water is ‘quality’. This factor has rarely been approached in the literature but is fundamental to public grievances concerning UOGD. Considering the type of issues highlighted by Vengosh et al. [14] and the language of GC15, which states that ‘the water required for each personal or domestic use must be safe, therefore free from micro-organisms, chemical substances and radiological hazards that constitute a threat to a person’s health’, the technique comes heavily under fire because it injects chemical substances during numerous phases of the process and leaves radiological contaminants above and below the surface free to pollute. Therefore, when UOGD production contaminates sub-surface and surface water resources it constitutes ‘a threat to a person’s health’ and to the environment that supports human health.

## 4. The Right to Water and ‘Fracking’

Referring states to WHO guidelines, GC15 states that standards should ensure ‘the safety of drinking water supplies through the elimination of, or reduction to a minimum concentration, of constituents of water that are known to be hazardous to health’. The General Comment also requires water to be of an acceptable colour, odour and taste for each personal or domestic use’. Taking Pennsylvania as an example, owing to the incidents and complaints that have been reported to the Pennsylvania Department of Environmental Protection (DEP), it is clear that actual incidents have occurred where quality issues undermined GC15 and, ultimately, the ICESCR in this context. In a major study of corporate violations, Inglis and Rumpler [70] conclude:

‘Drilling poses major risks to our water supplies, including potential underground leaks of toxic chemicals and contamination of groundwater. There are at least 243 documented cases of contaminated drinking water supplies across Pennsylvania between December 2007 and August 2014 due to fracking activities, according to the Pennsylvania Department of Environmental Protection (DEP)’ [70].

In fact, it is commonplace for oil and gas companies to be required bring in ‘replacement water supplies for residents, construct new drinking water wells, or otherwise modify their existing water wells’ in order to make the water potable following ‘fracking’ operations [70]. Some companies have been ‘cited’. For instance, in November 2012, Carrizo (Marcellus) LLC was cited (violation 653937) for failing to properly restore a contaminated drinking water supply following drilling operations in Forest Lake Township, Susquehanna County [70,71].

In cases like Forest Lake (and there are many), it is difficult to dispute that UOGD affects both the quantity and quality of water resources. Whereas legislation setting technical standards for drinking water quality is commonplace in countries around the world, we are reminded of the Halliburton Loophole that exempts fracking operations from the SDWA in the USA. The human rights aspect to fracking operations has arisen in response to what can be described as a belief that governments have failed to adequately protect citizens and their environment. Cooperation between the industry and its regulatory agencies—in ways that fail to respect human rights standards—has been perceived. The Halliburton Loophole is an illustration of a government and regulations failing to protect its citizens following corporate lobbying.

The nexus between human health, human rights and the environment and environmental rights has been succinctly forged over the last decade or so; in Europe (thus the UK) the establishment of the right to live in a healthy environment, born out of the Article 8 ECHR’s right to respect for one’s ‘private and family life and home’, is the embodiment of that profound connection between humans and their environment. Consequently, it should come as no surprise that human rights norms articulated in international declarations, conventions and treaties have come into question in the UOGD debate [4]. Traditionally human rights advocacy is sought when government agencies, legislatures and courts regularly fail to adequately protect the process, health, safety and other rights of their citizens. Therefore, the status of the right to water has come to the fore since the advent of the technology. The recognition of the right has become instrumental to protecting other inalienable rights.

Arguably, when human rights standards are applied, a ban (rather than regulation) is supported; especially as the emerging science suggests that the risks to public health are widespread and costly. Concerned citizens often see very good science—that suggests the risks are too high—brought into the public domain and ignored by decision-makers and a regulatory framework that is not geared to respond to new science, or in other words, in this case, an advanced industry in its infancy. In essence, it is evident that our laws are not prepared for either the new science that facilitates unconventional gas extraction or the scientific evidence that questions the safety and economic viability of UOGD. The negative impact data from all domestic contexts where there is an active unconventional industry strongly suggests that existing regulations are not fit for purpose, nor are the agencies or monitoring bodies, and nor the penalties imposed—the myriad of problems persist. Moreover, environment impact assessments do not routinely include a category that specifically covers fracking, and in some countries (e.g., in the UK) there is no fracking-specific regulation when the associated risks are quite simply not the same as conventional extraction, and existing technology is often found wanting when it comes to mitigating harm—nonetheless, many governments are aggressively promoting the technology. Accordingly, the right to water (within the human rights discourse), in conjunction with environmental law and water law, is poised to take centre stage in the ‘fracking debate’.

**Figure 2:**
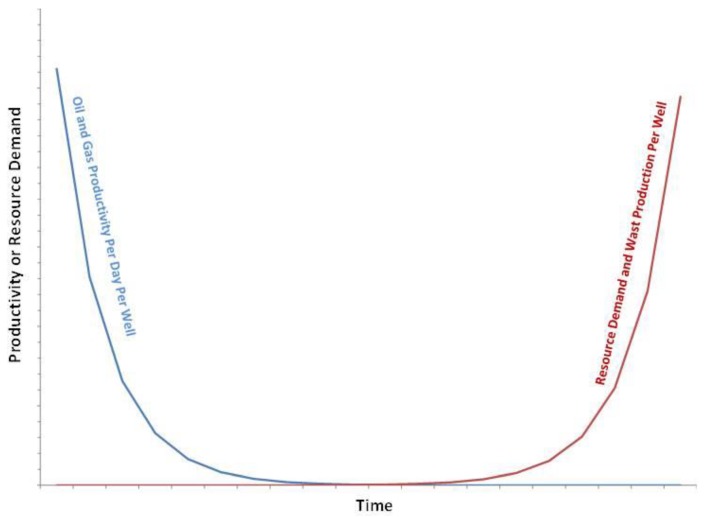
Theoretical relationship between resource demand and unconventional oil/gas production over time.

At the outset of the U.S. shale revolution there was quite a bit of discussion about “energy independence”, jobs, tax revenue, and short shrift was paid to concerns about issues such as air quality, community cohesion, ecological effects, and water quality and/or quantity. However, 5–15 years out from the beginning of the UOGD revolution we are seeing that realized tax revenue is often an order-of-magnitude less than projections, job creation has been replaced by job migration from shale play to shale play, and directional wells are proving short-lived (i.e., 85% declines in productivity from year 1 to year 2). The latter, along with expansion into less productive plays like the Antrim in Michigan, has resulted in the UOGD industry requiring more and more resources in the form of chemicals, sand [72], and water to stimulate production in the name of shareholder return—represented diagrammatically below (Figure 2):

Furthermore, the exponential increase in water demand, as well as ever longer laterals, has resulted in parallel rises in liquid and solid waste production. This waste increase has created tremendous stressors in states like Oklahoma and Ohio where “induced seismicity” or anthropogenic earthquake activity has increased from an average of 21 M3+ quakes between 1973 and 2008 to 659 of M3+ in 2014 alone according to the United States Geological Survey (USGS). These seismic events are not limited to smaller events; magnitudes exceeding 5.8 have been witnessed in Pawnee and Cushing, Oklahoma; and Cushing happens to be the site of the U.S.’s largest strategic commercial crude oil storage terminal prompting questions about whether the UGD’s excessive production of waste and demand for water may be compromising their own infrastructure and undercutting the notion that UGD will lead to “energy independence”.

**Figure 3:**
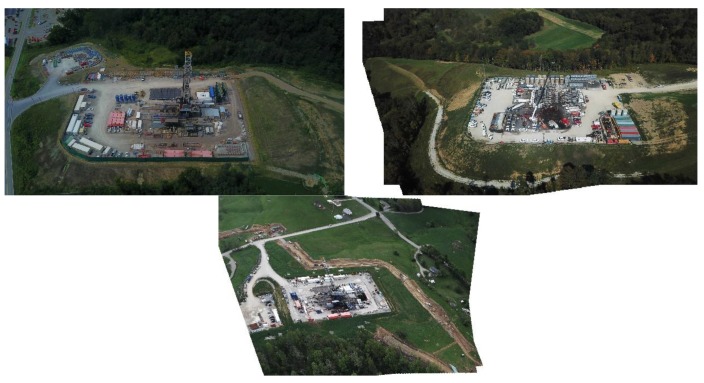
Examples of a typical hydraulic fracturing well pad in Eastern Ohio including a record breaking lateral pad at the Walleye Pad (Top-Left).

**Figure 4:**
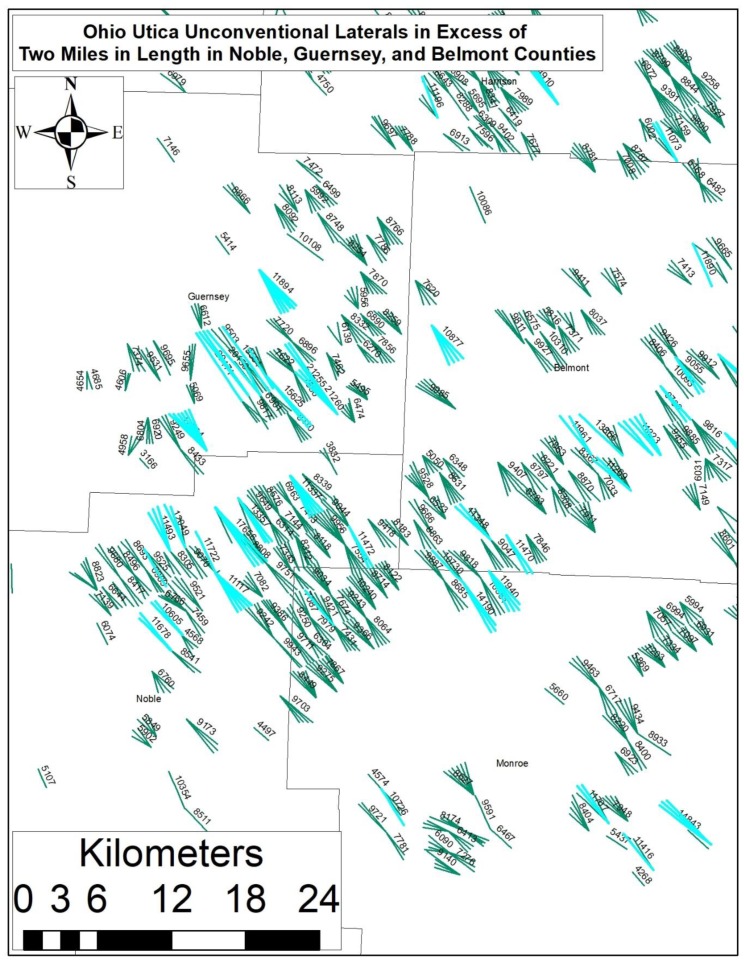
Examples of hydraulic fracturing lateral length in north-eastern Noble County, Ohio, including highlighted pads where laterals exceed 2 miles in length.

Examples of increasing resource demand include Chesapeake Energy’s ‘Propageddon’ lateral in Louisiana, which required more than 25 tons of frac sand (5–6 times the average amount of sand needed in a typical fracked lateral). From a water demand perspective, names like “Purple Hayes”, “Outlaw”, and “Walleye” have been given to “Super-Laterals” in Ohio and West Virginia where they exceed 17–20,000 feet in length, or 2.3–2.6 times average lateral length in these two states (Figure 3 and Figure 4). These laterals require more than 85 million gallons each, which translates into 4471 gallons per lateral foot (GPLF). To put this demand into some perspective, and assuming the average American uses 33,000 [73] gallons of water per year, this is roughly equivalent to the annual water demand of 2587 Americans.

**Figure 5:**
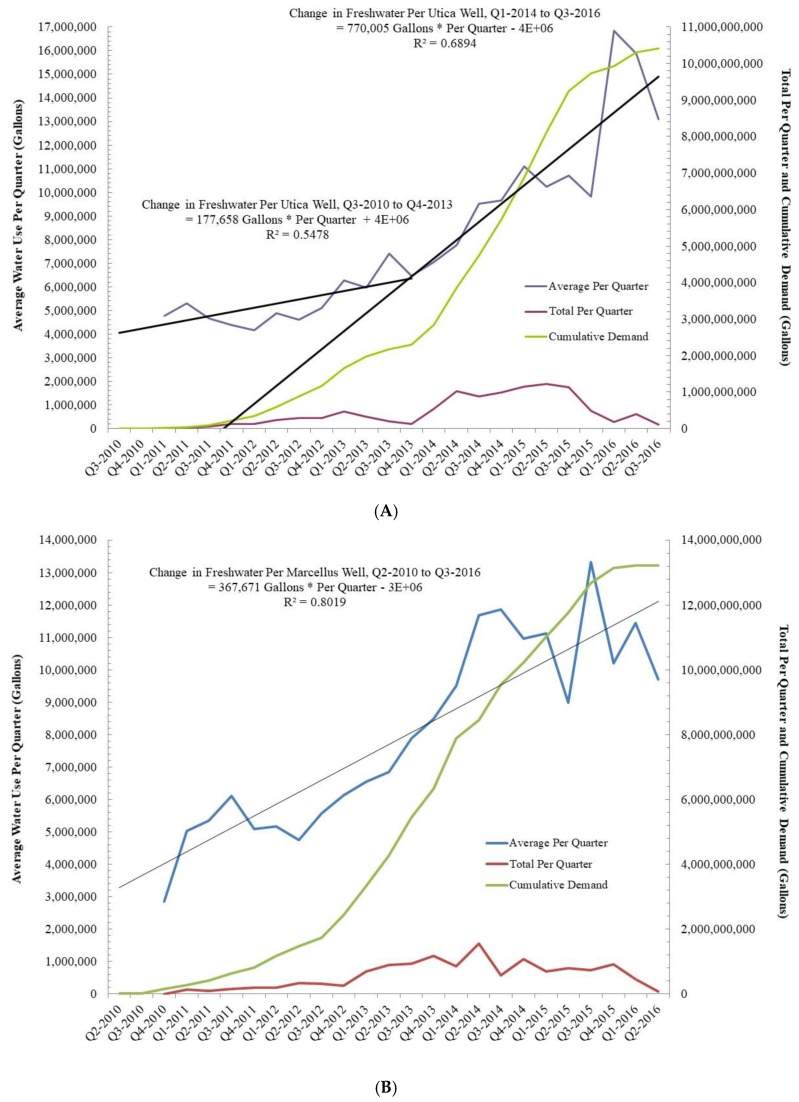
Ohio (**A**) and West Virginia (**B**) hydraulic fracturing freshwater demand in total and per well between Q3-2010 and Q3-2016.

Traditionally, Appalachian West Virginia and Ohio laterals require 970–1080 GPLF with demand growing at a rate of 11–22% per year (Figure 5). As an example of how much liquid—and potentially radioactive—waste is produced, we estimate that 11–12% of the freshwater used in the fracking process comes back to the surface as “brine” and must be disposed of in Class II Salt Water Disposal Injection Wells. Put another way an 85-million-gallon lateral would likely produce 9.8 million gallons of liquid waste, which is equivalent to the total amount of water in 15 Olympic sized swimming pools.

**Figure 6:**
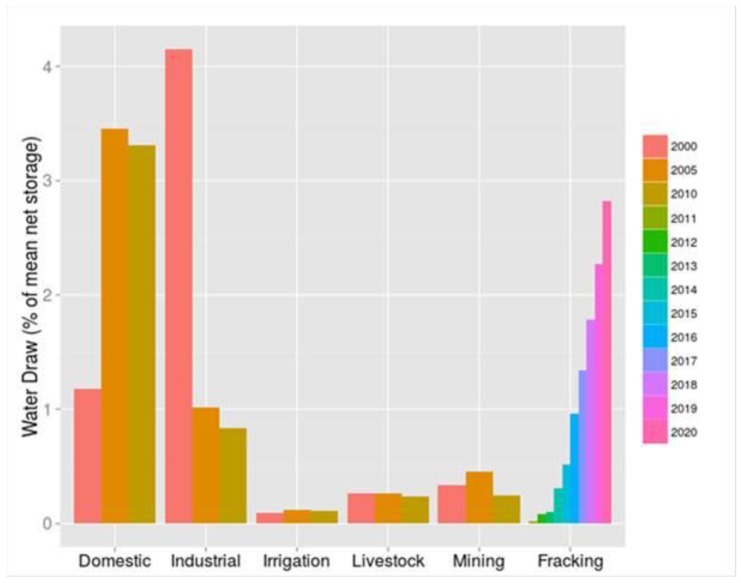
Freshwater demand across six sectors within the Muskingum River Watershed, south-east Ohio between 2000 and modelled out to 2020.

These recent developments call into question our existing resource-demand models, which estimate resource demand is increasing by 7–30% per year, while oil and gas production is declining by 85% per year per well. More importantly, these trends raise concerns about watershed ecological security and/or resilience, public water supply robustness, and the increasing importance of the UOGD’s water demand in modelling any given watershed’s redundancies in the face of less frequent and more intense precipitation events resulting from climate change (Figure 6).

**Figure 7:**
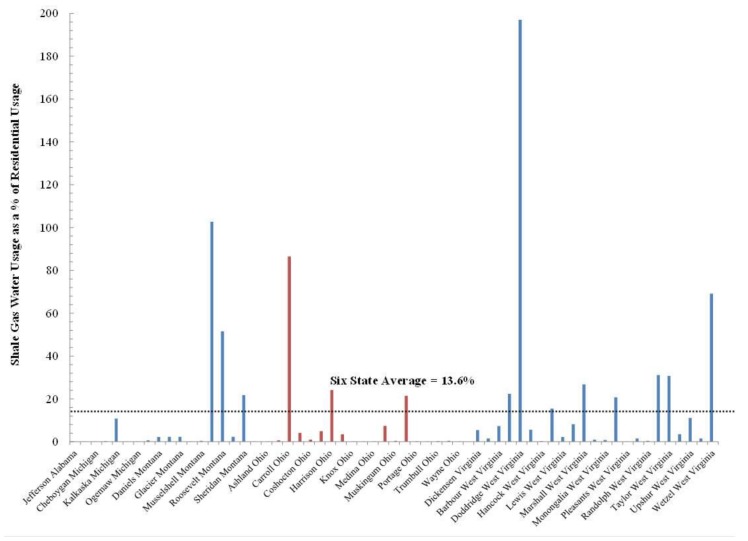
Hydraulic fracturing water demand across six states as a percent of annual residential water demand.

**Figure 8:**
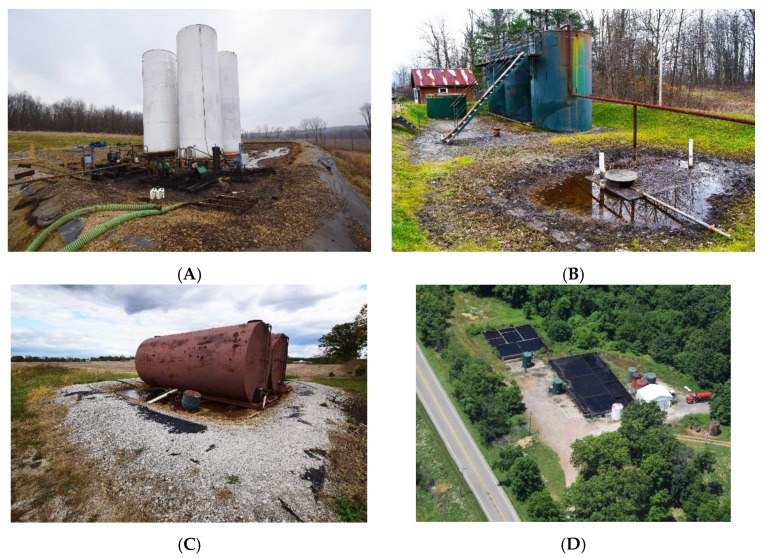
Class II injection wells—eastern Ohio. Danville (**A**), Knox (**B**), Morrow (**C**), and Stark (**D**) Counties.

At the present time UOGD’s water demand amounts to roughly 14% of residential water demand (Figure 7) but exceeds 65% in counties as geographically disparate as Carroll County, Ohio, Richland County, Montana, and Wetzel County, West Virginia. The most extreme example of how residential water demand is in conflict with UOGD’s demand is Doddridge County, West Virginia where UGD demand is nearly double that of residential demand. In Sposito’s [74] terms, these demands greatly exceed the “precautionary principle setting 20% of the natural runoff in a region as the upper limit of … consumptive use” by any one industry. The crux of the matter is that the industry is only charged $4.25–6.25 per thousand gallons, which only amounts to 0.25–0.28% of well-pad costs and is less than half of what residential users pay in the United States [75]. Furthermore, when the resulting liquid wastes are produced, the UOGD industry is charged $0.05–0.20 (This range is from Ohio alone and does not speak to a nationwide fee structure for UGD liquid waste disposal) per gallon disposed (e.g., 0.0044% of well-pad costs) across the country’s thousands of Class II injection wells with examples of such wells from eastern Ohio below (Figure 8).

Data from Colorado on how they price water for the fracking industry is even more concerning. Our research has found that Windsor Town Council sold Great Western Oil & Gas (GWOG) up to 65 acre ft (1 acre foot of water = 325,851 gallons) per year for the next 10 years. That contract can be renegotiated at that time. The town council reports that the cost that GWOG paid is $400/acre ft. That brings into the town of Windsor a meagre $26,000 annually should the entire 65 acre ft be taken, and it seems likely that GWOG will use the water rather quickly, based on past consumption and the requirements of the industry. Therefore, that brings the cost per gallon to $0.00122 or one tenth of one penny per gallon, not $0.01 but $0.00122.

**Figure 9:**
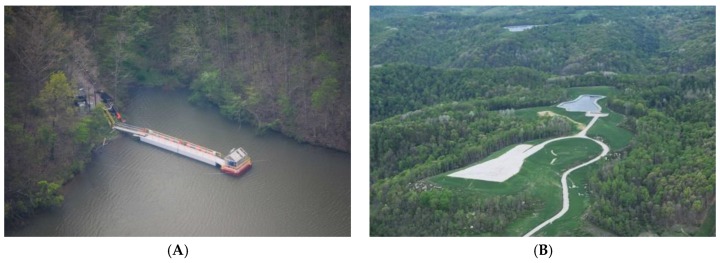
Typical unconventional oil and gas development (UOGD) freshwater infrastructure in south-east Ohio: Antero’s Barnesville Reservoir Pump (**A**); typical hydraulic fracturing well-pad and freshwater impoundment (**B**).

Such data highlights a crucial problem, there is no supply-side price signal demanding the UOGD industry reduce or stabilize their water demand per unit of energy produced. An additional issue concerns anecdotal evidence pointing towards the UOGD industry relying on highly fragile and ecologically critical 1st- and 2nd-order streams and reservoirs throughout Appalachia, when their demand cannot be met by documented water withdrawals agreements with conservancy districts (Figure 9). At the present time, research points to a 22–25% gap in our understanding of where this industry’s water demand is coming from; thus, leaving frontline communities and policy makers in the dark regarding how this known ‘unknown’ environmental externality will manifest in the coming years and decades.

Resource demand in the UOGD industry is directly related to the global price of oil and gas, with water demand increasing exponentially as the price of oil and gas declines. This forces the industry to rely on resources known to generate a disproportionate return-on-investment (ROI) relative to the price paid for the resources. As an example, the water demand inflection points we have documented in the Marcellus and Utica plays of southern Appalachia happened to coincide with a 50% decline in the global price of Brent crude and West Texas intermediate oil between Q1-2014 and the end of 2016.

## 5. Conclusions

Despite its widespread use in the United States for over a decade, hydraulic fracturing has only recently been scrutinized to determine the industry’s effects on human rights. Under the special procedures of the HRC, the Special Rapporteur on the human right to safe drinking water and sanitation, Catarina de Albuquerque, concluded her 2011 mission to the United States by outlining serious concerns over the effect of a range of polluting activities associated with the hydraulic fracturing process. She observed a distinct:

“policy disconnect ... between polluting activities and their ultimate impact on the safety of drinking water sources. The absence of integrated thinking has generated enormous burdens, including increased costs to public water systems to monitor and treat water to remove regulated contaminants and detrimental health outcomes for individuals and communities”.

[76]

There have been scores of scientific studies that have revealed water contamination due to fracking processes. Ingraffea’s review of the compliance reports from conventional and unconventional oil and gas wells drilled in Pennsylvania between 2000 and 2012 [9] revealed that casing/cement impairment is six times more likely to occur in unconventional wells than in conventional wells. Such flaws may result in cases of subsurface gas migration into the water supply, as has already occurred in the state. Indeed, published data demonstrates evidence of:

“Contamination of shallow aquifers with hydrocarbon gases … contamination of surface water and shallow groundwater from spills, leaks, and/or the disposal of inadequately treated shale gas wastewater… [and] accumulation of toxic and radioactive elements in soil or stream sediments near disposal or spill site s…from hydraulic fracturing throughout the United States”.

[14]

Qualitative data from Colorado has further revealed complaints of water contamination from residents living near fracking sites that are often intentionally misunderstood, assigned a different cause, or diluted by state regulatory bodies [77]. Recently, the Pennsylvania Department of Environmental Protection disclosed details of 243 cases in which fracking companies were found by state regulators to have contaminated private drinking water wells in the last four years [78]. In a much delayed survey of existing scientific literature on this topic (not a new data set), the U.S. Environmental Protection Agency found ‘scientific evidence that hydraulic fracturing activities can impact drinking water resources under some circumstances. The report identifies certain conditions under which impacts from hydraulic fracturing activities can be more frequent or severe:

- Water withdrawals for hydraulic fracturing in times or areas of low water availability, particularly in areas with limited or declining groundwater resources;
- Spills during the handling of hydraulic fracturing fluids and chemicals or produced water that result in large volumes or high concentrations of chemicals reaching groundwater resources;
- Injection of hydraulic fracturing fluids into wells with inadequate mechanical integrity, allowing gases or liquids to move to groundwater resources;
- Injection of hydraulic fracturing fluids directly into groundwater resources;
- Discharge of inadequately treated hydraulic fracturing wastewater to surface water; and
- Disposal or storage of hydraulic fracturing wastewater in unlined pits resulting in contamination of groundwater resources’ [79].

Cumulatively, the scientific literature, NGO and other policy reports and the vital testimony of local people indicate the likely impairment of the right to water for residents living near fracking sites. Even so, from the data presented here, we can see that perhaps the major issue regarding water use is the shifting of the resource from society to industry and the demonstrable lack of supply-side price signal that would demand the UOGD industry reduce or stabilize their water demand per unit of energy produced. Thus, in the U.S. context alone, there is considerable evidence that the human right to water is seriously undermined by the UOGD industry and, given its spread around the globe, this could soon become a global human rights issue.
